# Antioxidant Glutathione Analogues UPF1 and UPF17 Modulate the Expression of Enzymes Involved in the Pathophysiology of Chronic Obstructive Pulmonary Disease

**DOI:** 10.3390/cimb46030149

**Published:** 2024-03-12

**Authors:** Ingrid Oit-Wiscombe, Ursel Soomets, Alan Altraja

**Affiliations:** 1 Department of Pulmonology, University of Tartu, 50406 Tartu, Estonia; ioit@ut.ee; 2 Institute of Biomedicine and Translational Medicine, University of Tartu, 50411 Tartu, Estonia; 3 Centre of Excellence for Genomics and Translational Medicine, University of Tartu, 50411 Tartu, Estonia; 4 Lung Clinic, Tartu University Hospital, 50411 Tartu, Estonia

**Keywords:** COPD, glutathione, antioxidant, OS, systemic inflammation, glutathione analogues

## Abstract

Increased oxidative stress (OS) and systemic inflammation are key players in the pathophysiology of chronic obstructive pulmonary disease (COPD). We aimed to clarify the effects of synthetic glutathione (GSH) analogue peptides UPF1 and UPF17 on the mRNA levels of enzymes involved in systemic inflammation and GSH metabolism in peripheral blood mononuclear cells (PBMCs) from patients with acute exacerbation of COPD (AE-COPD) and stable COPD along with non-obstructive smokers and non-smokers. UPF1 and UPF17 increased the expression of enzymes involved in the formation of the antioxidant capacity: superoxide dismutase 1 (SOD1) and the catalytic subunit of glutamyl-cysteine ligase (GCLC) in patients with AE-COPD and stable COPD, but also in non-obstructive smokers and non-smokers. Similarly, both UPF1 and UPF17 increased the expression of inflammatory enzymes poly(ADP-ribose) polymerase-1 (PARP-1), dipeptidyl peptidase 4 (DPP4), and cyclooxygenase-2 (COX-2). Both UPF analogues acted in a gender-dependent manner by increasing the expression of certain anti-inflammatory (histone deacetylase 2 (HDAC2)) and GSH metabolism pathway (SOD1 and GSH reductase (GSR))-related enzymes in females and decreasing them in males. UPF1 and UPF17 are able to increase the expression of the enzymes involved in GSH metabolism and could serve as a lead for designing potential COPD therapies against excessive OS.

## 1. Introduction

Chronic obstructive pulmonary disease (COPD) causes remarkable systemic consequences that arise from triggering inflammation and oxidative stress (OS) in distant organs liaised by circulation [1,2,3]. The response by peripheral blood cells in COPD is less well understood, as there are little to no data on its modulation by antioxidants. Targeting OS and systemic inflammation could prove to be an effective way to improve survival and quality of life in these patients [1,3].

Pro-inflammatory enzymes cyclooxygenase-2 (COX-2), 5-lipoxygenase (5-LO), dipeptidyl peptidase 4 (DPP4), and leukotriene A_4_ hydrolase (LTA_4_H) and anti-inflammatory enzymes histone deacetylase 2 (HDAC2) and poly(ADP-ribose) polymerase-1 (PARP-1) have been demonstrated to be connected to COPD and underlying systemic inflammation [2,4,5,6]. Glutathione (GSH) is predominantly known as an antioxidant [7]. Increasing the level of intracellular GSH, through GSH metabolism pathway enzymes [superoxide dismutase 1 (SOD1), GSH synthetase (GSS), catalytic and modulatory subunits of glutamyl-cysteine ligase (GCLC and GCLM), GSH reductase (GSR) and GSH peroxidase (GPx)], could be useful in different clinical modes. Thanks to the diverse characteristics of GSH, many different GSH analogues with different properties have already been synthesized [8].

Two tetrapeptide analogues of GSH, named UPF1 (O-methoxy-L-tyrosinyl-γ- L-glutamyl-L-cysteinyl-glycine) and UPF17 (4-methoxy-L-tyrosinyl-α-L-glutamyl-L-cysteinyl-glycine), were chosen for this study to reveal their effect on the expression of antioxidative and proinflammatory enzymes. UPF1 and UPF17 are more hydrophobic than GSH and can therefore more strongly interact with plasma membrane and/or hydrophobic binding sites of different proteins [9,10]. It has been previously shown that UPF1 has a 60-fold higher antioxidative capacity and UPF17 has a 3000-fold higher antioxidative capacity compared to GSH [9,10]. Both of them are non-toxic to primary neuronal cultures [9,10]. In addition to having strong antioxidative capacities, Hansen et al. showed that the UPF1 mechanism is nuclear factor (erythroid-derived 2)-like 2 transcription factor (Nrf2)-mediated [11], which itself has been shown to have a connection with pro-inflammatory enzymes COX-2 and DPP4 [12,13] and anti-inflammatory enzyme HDAC2 [14]. On the basis of the knowledge that mRNA expression levels of enzymes associated with OS and inflammation have been influenced by the severity of COPD [2] and that we previously identified the cellular and molecular mechanisms associated with inflammation and impaired repair mechanisms that characterize the pathophysiology of COPD [15], we hypothesized that UPF1 and UPF17 have an impact on the mRNA expression levels of enzymes connected with OS and have an opposite effect on inflammatory enzymes providing a protective effect on the development of COPD in peripheral blood mononuclear cells (PBMCs) from male and female patients with COPD.

## 2. Materials and Methods

This study’s protocol was approved by the Ethics Committee on Human Research of the University of Tartu (protocol nr: 170/T-22) and all procedures were conducted according to the ethical standards of the World Medical Association Declaration of Helsinki: Ethical Principles for Medical Research Involving Human Subjects. All participants were recruited from the Department of Pulmonary Medicine, Tartu University Hospital, Tartu, and informed consent was obtained in written form from all individuals before entering this study.

### 2.1. Study Individuals

Patients diagnosed with a severe acute exacerbation of COPD (AE-COPD), defined as those requiring hospitalization according to the Global Initiative for COPD consensus document (GOLD 2022) [16], and patients diagnosed as having stable COPD of all categories and degrees of severity according to the GOLD 2022 [16] were included in this study. Patients were consecutively gathered in real-life scenarios, without bias toward age, gender, BMI, or stage of the disease. All patients with COPD had their post-bronchodilator FEV_1_/forced vital capacity (FVC) ratio below 0.7, were managed according to the refined symptom- and exacerbation-based ABCD assessment tool, and were followed up in accordance with the GOLD 2022 consensus report [16]. The exclusion criteria for patients with both stable COPD and AE-COPD included mechanical ventilation, hospitalization into an intensive care unit, unstable coronary artery disease, and the presence of active cancer. Healthy non-smokers and smokers with their lung function within a normal range were incorporated for reference. Current smokers were defined as persons who currently smoked ≥1 cigarette per day and non-smokers were defined as those who had never smoked or who had quit smoking for at least 6 months prior to this study. All study individuals were required to be without upper and lower respiratory tract infections (including acute bronchitis, bronchiolitis, and pneumonia) during this study and for at least 4 weeks before this study with the exception of patients with AE-COPD in whom acute respiratory infections other than pneumonia were allowed, provided that these infections had a causative relation to the current COPD exacerbation as judged by the treating physician. Spirometry [17] and lung diffusing capacity [18] measurements were performed in accordance with the American Thoracic Society/European Respiratory Society standards, whereas multi-ethnic and Finnish reference values were used for spirometry [19] and diffusing capacity [20] indices, respectively.

### 2.2. Extraction of PBMCs

PBMCs were separated from blood using BD Vacutainer CPT tubes (Becton Dickinson, Franklin Lakes, NJ, USA) within 2 h of collection, in which they were centrifuged at 1500× *g* for 21 min at 20 °C. Isolated PBMCs were washed twice with 10 mL phosphate-buffered saline and centrifuged at 300× *g* for 15 min and 10 min at 20 °C. Mononuclear cells were divided into three even amounts. One of them was cultivated in the presence of 0.5 mM UPF1, the second was cultivated in the presence of 0.5 mM UPF17, and the third portion was cultivated without UPF peptides, using RPMI-1640 medium (includes 10% fetal calf serum and 1% penicillin ⁄streptomycin). The incubation time was 12 h.

### 2.3. RNA Extraction from PBMCs and cDNA Synthesis

RNA was extracted from the mononuclear cells using the Trizol method according to the manufacturer’s protocol (Invitrogen, San Diego, CA, USA) and stored at −80 °C until cDNA synthesis.

cDNA was synthesized, by the reverse transcriptase reaction, from total RNA (250 ng) using the SuperScript III enzyme (Invitrogen, Carlsbad, CA, USA) according to the manufacturers’ instructions. The conditions for the reverse transcriptase reaction were incubation at 65 °C for 5 min, incubation at 0 °C for 1 min after that, and then synthesis at 50 °C for 90 min, followed by inactivation at 75 °C for 15 min; cDNA was stored at −80 °C until qRT-PCR.

### 2.4. Measurement of mRNA Expression

The gene expression levels were detected using the TaqMan-qRT-PCR method (ABI Prism 7900HT Sequence Detection System, Applied Biosystems by Life Technologies, Waltham, MA, USA) and using Hs00242302_m1 for PARP-1, Hs00231032_m1 for HDAC2, Hs00153133_m1 for COX-2, Hs00167536_m1 for 5-LO, Hs00175218 for DPP4, Hs00167536_m1 for LTA_4_H, Hs00166575 for SOD1, Hs00829989_gH for GPx, Hs00609286_m1 for GSS, Hs00167317_m1 for GSR, Hs00155249_m1 for GCLC, Hs00157694_m1 for GCLM, and hypoxanthine phosphoribosyl- transferase-1 (HPRT-1) as a housekeeper gene (both from Applied Biosystems by Life Technologies, Waltham, MA, USA). All reactions were carried out in quadruplicates. The comparative Ct method (ΔCt value) was used for the quantification of mRNA, where the amount of target transcript was normalized to the level of endogenous housekeeper gene HPRT-1.

### 2.5. Statistical Analysis

The effect of the GSH analogues on the expression of the enzymes was analyzed using a linear mixed model for repeated measures and IBM SPSS Statistics (version 29, Chicago, IL, USA). Data were adjusted for age, gender, pack-years of smoking, the GOLD 2022 group [16], and COPD exacerbation status. Missing enzyme expression level data (12 variables) were populated by multiple imputation with random numbers within the range of measured data, leading to 5 new datasets. *p*-values < 0.05 were considered to indicate statistical significance.

## 3. Results

A total of 116 subjects were recruited. Non-obstructive non-smokers, non-obstructive smokers, and GOLD 2022 A-D COPD groups [16] were used to compare the enzymes involved in systemic inflammation and GSH metabolism mRNA expression levels in PBMCs (Table 1).

**Figure 1:**
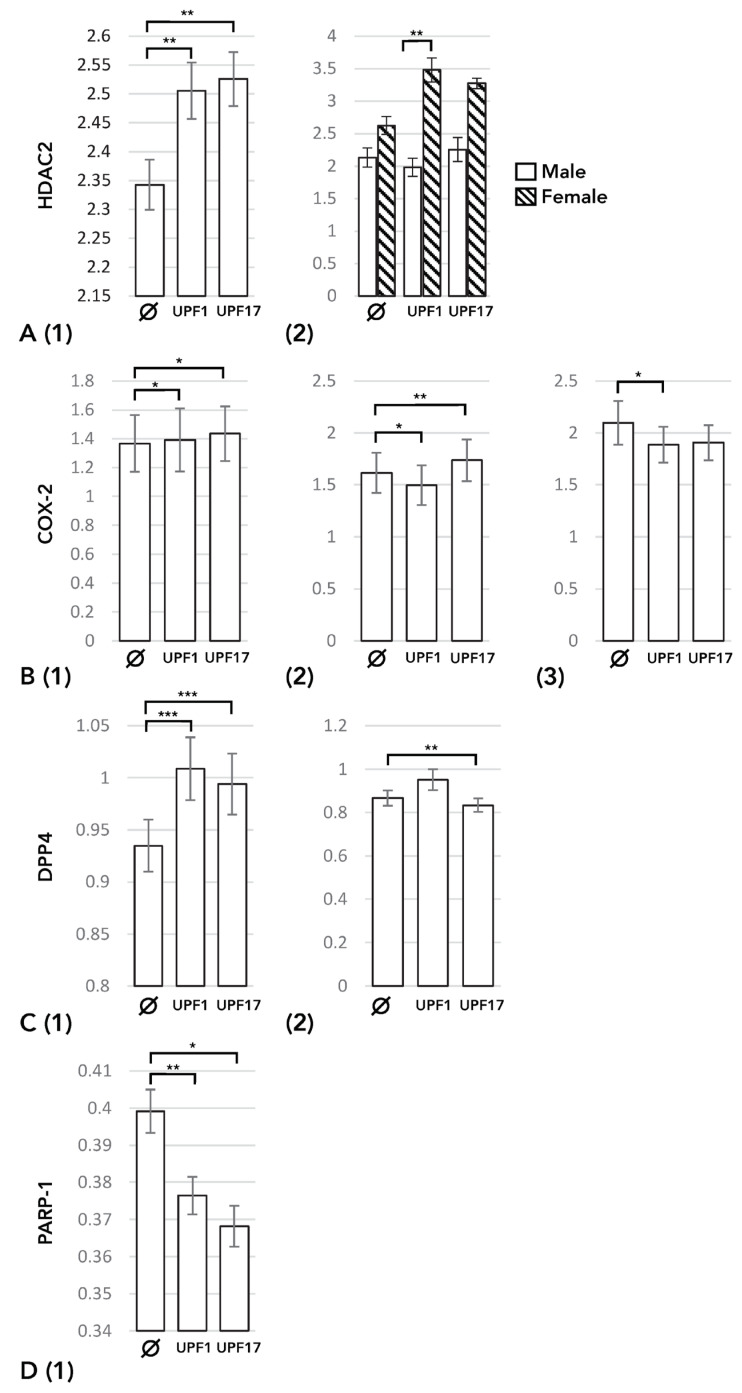
The effect of glutathione (GSH) analogues UPF1 and UPF17 on the levels of expression of the mRNA of the enzymes involved in inflammation analyzed using a linear mixed model for repeated measures. Data are adjusted for age, gender, and body mass index (BMI). (**A**) UPF1 and UPF17 effect on the mRNA expression level of histone deacetylase-2 (HDAC2) (1) in all individuals and (2) in patients who had never smoked, using both male and female subjects. (**B**) The effect of UPF1 and UPF17 on the mRNA expression level of cyclooxygenase-2 (COX-2) (1) in patients with ongoing acute COPD exacerbation, (2) in patients with a history of at least two moderate or at least one severe exacerbation within the last year, and (3) in COPD patients who had quit smoking at least 6 months prior to this study. (**C**) The effect of UPF1 and UPF17 on the mRNA expression level of dipeptidyl peptidase 4 (DPP4) (1) in all individuals and (2) in non-obstructive individuals and COPD patients who had quit smoking at least 6 months prior to this study. (**D**). The effect of UPF1 and UPF17 on the mRNA expression level of poly(ADP-ribose) polymerase-1 (PARP-1) (1) in all individuals analyzed together. * *p* ≤ 0.05, ** *p* ≤ 0.01, *** *p* ≤ 0.001 vs. baseline (∅).

**Figure A1:**
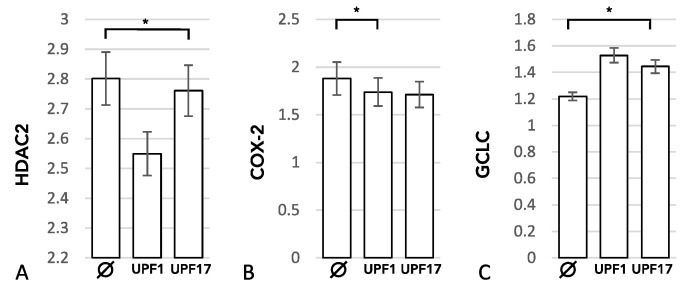
The effect of glutathione (GSH) analogues UPF1 and UPF17 on the levels of expression of the mRNA of the enzymes involved in GSH metabolism and inflammation, analyzed using a linear mixed model for repeated measures. Data are adjusted for age, gender, and body mass index (BMI). UPF1 and UPF17 effect on the mRNA expression level of (**A**) histone deacetylase-2 (HDAC2) in patients with ≥2 moderate or ≥1 severe AE-COPD within the last year, (**B**) cyclooxygenase-2 (COX2) in COPD patients and non-obstructive individuals who had never smoked or had quit at least 6 months prior, (**C**) glutamate-cysteine ligase catalytic subunit (GCLC) in all individuals. * *p* ≤ 0.05 vs. baseline (∅).

UPF1 and UPF17 significantly increased HDAC2 expression amongst all subjects (*p* = 0.001) (Figure 1A, left panel; Table 2). However, UPF17 decreased HDAC2 expression amongst patients with ≥2 moderate or ≥1 severe AE-COPD within the last year (*p* = 0.01) (Figure A1A). Amongst individuals who had never smoked, the effect of UPF1 was the opposite between males and females, with a decrease amongst the former and an increase amongst the latter (*p* = 0.009) (Figure 1A, right panel).

In patients with an ongoing AE-COPD, UPF1 and UPF17 significantly increased COX-2 expression (*p* = 0.027 and *p* = 0.049, respectively) (Figure 1B, left panel; Table 3). UPF17 increased (*p* = 0.007) but UPF1 decreased (*p* = 0.018) COX-2 expression amongst patients with a history of ≥2 moderate or ≥1 severe AE-COPD within the last year (Figure 1B, middle panel). Additionally, UPF1 decreased COX-2 expression amongst the COPD patients who had quit smoking at least 6 months ago (*p* = 0.049) (Figure 1B, right panel). A similar decrease in COX-2 expression was evident, when both COPD patients and non-obstructive individuals, who had never smoked or had quit at least 6 months ago, were analyzed together (*p* = 0.037) (Figure A1B).

Both UPF1 and UPF17 increased DPP4 expression amongst all participants (*p* < 0.001 and *p* < 0.001, respectively) (Figure 1C, left panel; Table 2). On the contrary, UPF17 decreased DPP4 expression amongst ex-smokers, who had quit smoking at least 6 months ago (*p* = 0.006) (Figure 1C, right panel).

UPF1 and UPF17 decreased PARP-1 expression amongst all participants (*p* = 0.001 and *p* = 0.039, respectively) (Figure 1D; Table 2).

**Figure 2:**
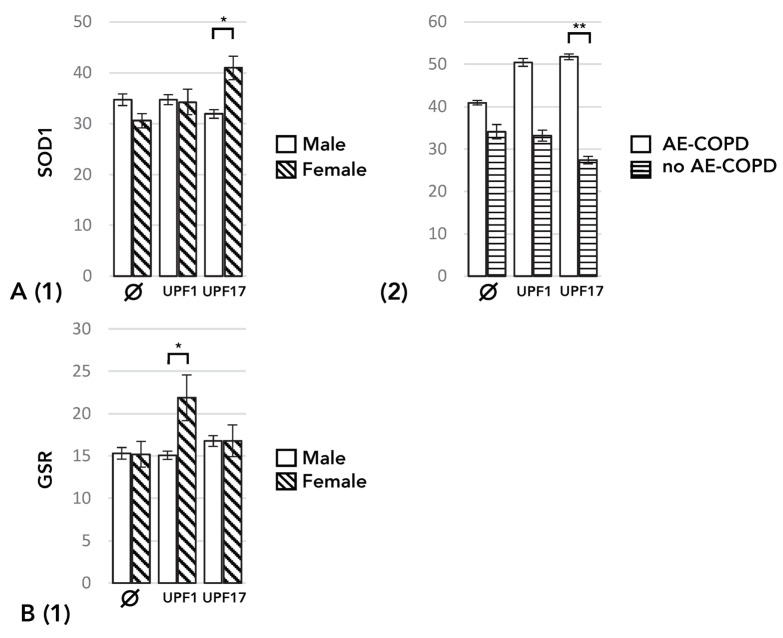
The effect of GSH analogues UPF1 and UPF17 on the levels of expression of the mRNA of the enzymes involved in GSH metabolism analyzed using a linear mixed model for repeated measures. Data are adjusted for age, gender, and body mass index (BMI). (**A**) The effect of UPF1 and UPF17 on the mRNA expression level of superoxide dismutase-1 (SOD1) (1) with the inclusion of all individuals and comparing males and females and (2) with the inclusion of all current smokers, comparing patients with current AE-COPD and those without AE-COPD. (**B**) The effect of UPF1 and UPF17 on the mRNA expression level of glutathione reductase (GSR) (1) with the inclusion of all individuals and comparing males and females. * *p* ≤ 0.05, ** *p* ≤ 0.01 vs. baseline (∅).

SOD1 expression was only influenced by UPF17 in a gender- and AE-COPD-dependent manner as follows. UPF17 decreased SOD1 expression in males and increased it in females, when all study individuals were included (*p* = 0.022) (Figure 2A, right panel). Amongst the current smokers, SOD1 expression was increased by UPF17 amongst the patients with AE-COPD and decreased among patients without it (*p* = 0.001) (Figure 2A, right panel). Contrary to this, in SOD1, GSR expression was only influenced by UPF1 and in a gender-dependent fashion: UPF1 increased the GSR mRNA expression level in females and decreased it in males, when all study individuals were included (*p* = 0.046) (Figure 2B).

The expression of GCLC was significantly influenced only by UPF17, which showed an increase, when all participants were analyzed (*p* = 0.012) (Figure A1C; Table 2).

## 4. Discussion

Both GSH analogues, UPF1 and UPF17, decreased the mRNA expression level of PARP-1 amongst all individuals. We have previously shown that UPF17 had a significant inhibitory effect on the PARP-1 mRNA expression level in PBMCs from patients with COPD compared to that in non-obstructive individuals [2]. The increased expression of PARP-1, which is generally responsible for more than 90% of the cellular poly(ADP-ribosyl)ation capacity, may be protective against OS-related DNA damage [21]. On the other hand, when PARP-1 is overexpressed, its inhibition would be metabolically favorable [21,22]. Modest inhibition of PARP-1 expression can have anti-inflammatory effects, but inhibiting PARP-1 to a greater degree in return might reduce cellular DNA repair and can thus be deleterious in most circumstances [2,23]. 

Both UPF peptides increased the DPP4 expression level amongst all patients. The DPP4 gene is related to the signaling pathway, which in turn is related to T-lymphocyte activation and antigen-reporting cell and T-cell interactions [24]. We have previously shown that the expression of DPP4 is downregulated in PBMCs in COPD patients with more severe airflow obstruction and AE-COPD history [15]. Seys et al. showed that DPP4 mRNA expression was elevated in the lung tissue of patients with COPD compared to smokers and never-smokers without an airflow limitation [6]. Increasing the systemic expression of DPP4 may help patients with COPD to mobilize their systemic inflammatory response. It might be of interest that UPF17 decreased DPP4 expression amongst ex-smokers who had quit at least 6 months prior to this study. This could be explained by inhibiting systemic inflammation through DPP4, when removing immense cigarette smoke (CS)-induced systemic OS. This could even have beneficial effects on lung function [25]. However, as has been formerly shown, the use of DPP4 inhibitors can lead to worsening heart failure in patients with established cardiovascular disease or metabolic abnormalities [26]. Hence, decreasing DPP4 might not be unequivocally favorable in COPD patients with cardiovascular or metabolic comorbidities.

UPF1 as well as UPF17 elevated COX-2 expression in patients with AE-COPD. Elevated COX-2 expression has been associated with several chronic inflammatory diseases such as COPD [27]. Since COX-2 is the rate-limiting enzyme in prostanoid synthesis, its upregulation is expected to lead to an increase in the production of downstream prostanoid products [28]. Eicosanoids are potent inflammatory mediators that are produced from arachidonic acid by cyclooxygenases and lipoxygenase (LO) [29]. LTA_4_H is a major enzyme of the 5-LO pathway [30]. Interestingly, in our current study, neither UPF1 nor UPF17 had an influence on LTA_4_H expression.

The two GSH analogues UPF1 and UPF17 increased HDAC2 expression amongst all patients. Increasing HDAC2 expression/activity can restore the failure to suppress OS-induced pro-inflammatory cytokine production [31]. Interestingly, when COPD patients and non-obstructive smokers were analyzed separately as a subgroup, UPF1 increased the HDAC2 expression level in female individuals but decreased it in males. It has been previously shown that HDAC epigenetic factors, at least in part, regulate liver recovery rates differently in male and female mice, which might be caused by an initially lower expression of HDAC1 and HDAC2 in female mice [32]. Zhao et al. showed that HDAC2 expression in mice might be sex-hormone-influenced [33]. UPF1′s ability to differently influence HDAC2 expression in female and male individuals in human PBMCs in the present study may be related to different regulation of inflammatory genes. As far as we are aware, this was the first study that investigated gender differences in HDAC2 in human PBMCs and further studies are needed before substantial conclusions can be made. UPF17 also decreased the HDAC2 mRNA expression level in patients with a history of ≥2 moderate or ≥1 severe exacerbation within the last year. COPD and AE-COPD are mediated by the increased expression of multiple inflammatory genes, many of which are regulated by the acetylation of core histones around which DNA is wound. Conversely, these activated genes are switched off by deacetylation of these histones, resulting in suppression of the inflammatory gene expression by HDACs [34]. CS-induced OS is not only associated with an accumulation of reactive oxygen species (ROS) but also with a depletion of GSH, which together inhibit HDACs’ activity [35]. This in turn leads to increased acetylation of histones and thus DNA uncoiling which can cause increased expression of pro-inflammatory genes [35]. We have recently shown that HDAC2 expression in PBMCs correlates with the severity of airflow limitation in patients with COPD [15]. Previously, it has also been shown by others that HDAC activity is decreased in patients with COPD in the peripheral lung and in alveolar macrophages in particular [34,36]. Inhibiting HDAC2 in a colorectal cancer cell line could act as a potential therapy because of exerting antiproliferative and antimigratory effects [37]. This knowledge cannot, however, be directly transposed to COPD patients, as increasing HDAC2 expression is known to suppress inflammatory gene expression to balance out chronic systemic inflammation [38].

Amongst current smokers, UPF17 increased SOD1 mRNA expression in patients with AE-COPD and decreased it in patents without it. We have shown that SOD1 expression increases in parallel with a decline in lung function [15]. SOD1 is a key factor in eliminating the superoxide radicals and its expression has been studied in different inflammatory diseases [39]. The importance of antioxidants for protection against CS has been shown before [40]. Foronjy et al. showed that transgenic mice with an overexpression of SOD1 prevented the formation of smoke-induced emphysema [40]. Inversely, inhibiting SOD1 could increase ROS levels, which in turn could increase DNA damage and, consequently, activate PARP-1 [41]. Upregulation of PARP-1 has been linked to the enhanced anti-apoptotic property of tumors [42]. Similarly to that of SOD1, the expression of another enzyme in the GSH synthesis pathway, GCLC, was increased by UPF17 amongst all patients. The GSH tripeptide is synthesized from glutamate, cysteine, and glycine. Glutamate-cysteine ligase (GCL), which is composed of a catalytic (GCLC) and a modifier (GCLM) subunit, catalyzes the rate-limiting reaction by converting cysteine and glutamate to *γ*-glutamyl-cysteine, and from this, with glycine, GSH is formed [43,44]. Similarly, Hansen et al. showed that UPF1 increases intracellular GSH levels by increasing Nrf2-mediated GCLC mRNA expression levels in the K562 human cell line [11]. Altraja et al. showed that UPF1 is able to entirely restore the intracellular GSH levels in human bronchial epithelial cells under oxidative stress caused by CS condensate [45]. Increasing antioxidants, like GSH in PBMCs, can prevent smoke-induced systemic inflammation.

In a gender-dependent fashion, UPF17 decreased SOD1 expression in males and increased it in females. Contrary to that in SOD1, UPF1 decreased the GSR mRNA expression level in males and increased it in female individuals. As far as we are aware, we are the first to show gender-dependent behavior of SOD1 and GSR in PBMCs. Frutiger et al. showed a specific gender-dependent alteration of SOD1 concentration in the cerebrospinal fluid of patients with amyotrophic lateral sclerosis [46]. Our finding supports the hypothesis of Maury et al. that there are clinical and physiological differences between male and female patients with COPD [47]. Why antioxidants SOD1 and GSR are differently influenced in PBMCs in male and female subjects needs to be further studied. From a therapeutic perspective, the notion of increasing antioxidants intercepts ROS and therefore provides an anti-inflammatory effect [48], whereas decreasing antioxidants increases ROS-induced OS and pro-inflammatory immunoinflammatory responses [49].

The limitations of this study include small cohort sizes in certain subgroups, e.g., female patients with COPD, GOLD 2022 group C, that could reduce the magnitude of the differences found. Secondly, this was a single-center study and the involvement of a wider population might have increased the significance of our results. Collaborating with multiple centers would have provided us with more diverse population coverage and increased generalizability. Lastly, the treatment part of our study was performed ex vivo and due to this, it might not fully replicate the exact physiological condition. Future studies in vivo could help to better understand the clinical relevance and potential therapeutic efficacy of UPF1 and UPF17. The gene expression data should be confirmed by functional proteomics or enzymatic analysis to elucidate the levels of the cytokines involved in the respective cascades or clarify the ability to scavenge ROS. To counterbalance this, the strengths of our current research include the large number of enzymes measured: six inflammatory enzymes and six anti-OS enzymes.

In conclusion, our current study shows that the investigated UPF peptides, despite exerting antioxidant properties, increased the expression of certain pro-inflammatory enzymes. Therefore, these peptides could not possibly be used directly themselves, but could nevertheless serve as a lead for designing potential COPD therapies against excessive OS. 

## Figures and Tables

**Table 1:** Characteristics of the individuals included to the measurement of the mRNA expression of enzymes implicated in systemic inflammation and glutathione (GSH) metabolism in peripheral blood mononuclear cells. Patients with exacerbation of chronic obstructive pulmonary disease (COPD), as well as those with a stable COPD of all categories, were diagnosed in accordance with the Global Initiative for COPD (GOLD) consensus document 2022 [16].

Characteristics	Non-Smoking Controls (*n* = 10)	Non-Obstructive Smokers (*n* = 15)	COPD			
			GOLD A (*n* = 25)	GOLD B (*n* = 30)	GOLD C (*n* = 4)	GOLD D (*n* = 32)
Age	64.0 ± 3.4	61.1 ± 2.9	69.0 ± 2.5	67.1 ± 2.0	80.0 ± 2.7	71.7 ± 1.6
Male	7 (70%)	9 (60%)	21 (84%)	29 (97%)	4 (100%)	31 (97%)
BMI	25.7 ± 1.6	27.0 ± 1.6	25.8 ± 1.0	23.4 ± 0.8	22.1 ± 0.6	24.0 ± 0.9
Smoking (pack-years)	-	35.7 ± 2.6	40.4 ± 3.8	39.5 ± 3.6	45.0 ± 5.0	43.5 ± 4.4
Current smoker	-	10 (67%)	17 (68%)	16 (53%)	0 (0%)	13 (41%)
Patients with an ongoing AE-COPD	-	-	2 (8%)	7 (23%)	3 (75%)	32 (100%)
Patients with a history of ≥2 moderate or ≥1 severe AE-COPD in the past year	-	-	-	-	4 (100%)	32 (100%)
Smoking cessation amongst ex-smokers (years ago)	-	6.8 ± 3.5	12.1 ± 3.4	9.0 ± 2.5	7.6 ± 3.1	7.9 ± 1.9
FEV_1_ % predicted	97.6 ± 4.5	82 ± 4.5	53.6 ± 4.7	41.1 ± 3.4	34.8 ± 3.0	30.0 ± 1.8
Absolute decline in FEV_1_ % over years (%/year) *	0.1 ± 0.1	0.6 ± 0.1	1.4 ± 0.4	1.6 ± 0.2	1.2 ± 0.1	1.6 ± 0.1
PEF % predicted	99.5 ± 6.8	80.6 ± 4.1	43.7 ± 4.3	35.1 ± 2.9	27.8 ± 5.5	27.3 ± 1.6
FVC % predicted	96.8 ± 4.7	79.9 ± 4.7	70.8 ± 4.8	59.2 ± 3.8	53.8 ± 7.8	46.7 ± 2.7
FEV_1_/FVC %	81.9 ± 1.2	82.3 ± 1.6	62.7 ± 1.9	60.9 ± 1.6	55.8 ± 2.6	52.5 ± 1.5
K_CO_	1.2 ± 0.1	1.2 ± 0.1	1.0 ± 0.1	0.8 ± 0.1	0.6 ± 0.0	0.6 ± 0.1
K_CO_ %	93.4 ± 4.8	80.3 ± 8.9	76.4 ± 7.5	56.6 ± 5.6	48.8 ± 0.0	40.5 ± 9.5
D_LCO_	6.3 ± 0.6	5.6 ± 0.8	4.8 ± 0.6	3.5 ± 0.4	2.8 ± 0.0	2.8 ± 0.6
D_LCO_ %	77.3 ± 9.0	61.6 ± 7.4	54.4 ± 5.3	38.7 ± 3.8	30 ± 0.0	31.3 ± 6.1
T_LC_	5.3 ± 0.3	4.82 ± 0.3	4.8 ± 0.3	4.6 ± 0.2	4.8 ± 0.0	4.6 ± 0.3
T_LC_ %	84.9 ± 5.8	79.2 ± 3.4	73.9 ± 2.1	68.0 ± 2.5	63.4 ± 0.0	67.4 ± 3.2

Data are presented as mean ± SEM or n (%). * Annual change in FEV_1_% predicted after the age of 25, assuming FEV_1_% was 100% at the age of 25 years. AE-COPD—acute exacerbation of chronic obstructive pulmonary disease, BMI—body mass index, D_LCO_—diffusing capacity of the lungs for carbon monoxide, FEV_1_—forced expiratory volume, FVC—forced vital capacity, K_CO_—carbon monoxide transfer coefficient, PEF—peak expiratory flow, SEM—standard error of mean, T_LC_—total lung capacity.

**Table 2:** The effect of GSH analogues UPF1 and UPF17 on the levels of expression of the mRNA of the enzymes involved in GSH metabolism and inflammation in non-obstructive non-smokers, non-obstructive smokers, patients with an acute exacerbation of COPD, and those with a stable COPD of all categories in accordance with the GOLD consensus document 2022 [16]. Analyses were conducted with the use of a linear mixed model for repeated measures adjusted for age, gender, and body mass index. Data are presented as mean ± SEM. *p*-values indicate significance for changes by UPF1 and UPF17, respectively.

Enzyme	Baseline	UPF1	*p* Value	UPF17	*p* Value
Pro- and anti-inflammatory enzymes					
COX-2	1.46 ± 0.28	1.46 ± 0.28	0.05	1.43 ± 0.27	0.19
5-LO	11.43 ± 1.05	10.56 ± 0.96	0.69	10.27 ± 0.89	0.62
DPP4	0.88 ± 0.06	0.94 ± 0.07	<0.001	0.93 ± 0.07	<0.001
LTA_4_H	4.79 ± 0.41	5.81 ± 0.59	0.88	5.62 ± 0.53	0.80
HDAC2	2.33 ± 0.11	2.45 ± 0.12	0.03	2.52 ± 0.11	0.01
PARP-1	0.40 ± 0.01	0.37 ± 0.01	0.001	0.36 ± 0.01	0.03
GSH metabolism enzymes					
SOD-1	34.00 ± 2.45	33.98 ± 2.17	0.89	32.43 ± 1.98	0.91
GSS	4.06 ± 0.18	3.57 ± 0.17	0.59	3.79 ± 0.19	0.29
GCLC	1.22 ± 0.08	1.30 ± 0.13	0.14	1.30 ± 0.12	0.01
GCLM	0.16 ± 0.02	0.17 ± 0.02	0.72	0.16 ± 0.02	0.66
GSR	14.12 ± 1.49	15.10 ± 1.14	0.94	15.32 ± 1.35	0.95
GPx	300.27 ± 24.25	284.20 ± 26.02	0.99	327.93 ± 31.97	0.94

5-LO—5-lipoxygenase, COX-2—cyclooxygenase-2, DPP4—dipeptidyl peptidase 4, GCLC—catalytic subunit of glutamyl-cysteine ligase (GCL), GCLM—modulatory subunits of GCL, GPx—glutathione (GSH) peroxidase, GSR—GSH reductase, GSS—GS synthetase, HDAC2—histone deacetylase 2, LTA_4_H—leukotriene A_4_ hydrolase, PARP-1—poly(ADP-ribose) polymerase-1, SEM—standard error of mean, SOD1—superoxide dismutase 1.

**Table 3:** The effect of GSH analogues UPF1 and UPF17 on the levels of expression of the mRNA of the enzymes involved in GSH metabolism and inflammation in patients with ongoing exacerbation of chronic obstructive pulmonary disease diagnosed in accordance with the GOLD consensus document 2022 [16]. Analyses were conducted with the use of a linear mixed model for repeated measures adjusted for age, gender, and body mass index. Data are presented as mean ± SEM.

Enzyme	Baseline	UPF1	*p* Value	UPF17	*p* Value
Pro- and anti-inflammatory enzymes					
COX-2	1.07 ± 0.52	1.03 ± 0.50	0.03	0.90 ± 0.41	0.05
5-LO	12.87 ± 2.63	12.84 ± 2.10	0.79	14.02 ± 2.33	0.70
DPP4	0.81 ± 0.08	0.79 ± 0.09	0.33	0.62 ± 0.09	0.10
LTA_4_H	4.46 ± 0.71	5.53 ± 0.93	0.91	5.17 ± 0.65	0.74
HDAC2	2.56 ± 0.28	2.45 ± 0.19	0.16	2.69 ± 0.28	0.07
PARP-1	0.45 ± 0.03	0.40 ± 0.03	0.76	0.39 ± 0.03	0.13
GSH metabolism enzymes					
SOD-1	31.60 ± 3.91	34.30 ± 6.13	0.86	36.11 ± 5.37	0.89
GSS	4.21 ± 0.55	3.49 ± 0.36	0.40	3.12 ± 0.38	0.15
GCLC	1.12 ± 0.13	1.23 ± 0.15	0.36	1.15 ± 0.16	0.14
GCLM	0.11 ± 0.02	0.21 ± 0.08	0.24	0.21 ± 0.03	0.84
GSR	11.40 ± 2.30	15.62 ± 3.19	0.86	11.55 ± 2.60	0.91
GPx	333.92 ± 53.13	310.81 ± 60.76	0.99	311.54 ± 51.74	1.00

5-LO—5-lipoxygenase, COX-2—cyclooxygenase-2, DPP4—dipeptidyl peptidase 4, GCLC—catalytic subunit of glutamyl-cysteine ligase (GCL), GCLM—modulatory subunits of GCL, GPx—glutathione (GSH) peroxidase, GSR—GSH reductase, GSS—GS synthetase, HDAC2—histone deacetylase 2, LTA_4_H—leukotriene A_4_ hydrolase, PARP-1—poly(ADP-ribose) polymerase-1, SEM—standard error of mean, SOD1—superoxide dismutase 1.

## Data Availability

Data are available upon request by mailing the corresponding author.
